# Differences Between Brachial-Ankle Pulse Wave Velocity and Lower Leg Circumference Ratio in Patients With and Without Type 2 Diabetes Mellitus

**DOI:** 10.7759/cureus.66902

**Published:** 2024-08-14

**Authors:** Tomoki Furuya, Shinji Kitahama, Daichi Yamashiro, Keigo Hinakura, Hajime Tamiya, Susumu Ogawa, Yuma Tamura, Tomoya Takahashi, Takanori Yasu, Hiroyuki Suzuki

**Affiliations:** 1 Research Team for Social Participation and Healthy Aging, Tokyo Metropolitan Institute for Geriatrics and Gerontology, Itabashi, JPN; 2 Department of Cardiovascular Medicine and Nephrology, Dokkyo Medical University Nikko Medical Center, Nikko, JPN; 3 Department of Rehabilitation and Physical Therapy, Igaku Academy, Saitama, JPN; 4 Department of Endocrinology, Kawatsuru Plaza Clinic, Kawagoe, JPN; 5 Research Team for Social Participation and Healthy Aging, Tokyo Metropolitan Institute for Geriatrics and Gerontology, Tokyo, JPN; 6 Department of Physical Therapy, Faculty of Rehabilitation, Niigata University of Health and Welfare, Niigata, JPN; 7 Department of Rehabilitation, Dokkyo Medical University Nikko Medical Center, Nikko, JPN

**Keywords:** simplified test, soleus muscle, sarcopenia, ba-pwv, arterial stiffness, type 2 diabetes, lower leg circumference ratio

## Abstract

Background

Vascular endothelial dysfunction in type 2 diabetes mellitus (T2DM) patients causes atherosclerosis and microvascular damage. This study investigated the relationship between leg circumference and arterial stiffness in patients with T2DM compared to non-T2DM individuals.

Methods

Data from two studies were combined to form T2DM (T2DM group) and non-T2DM (N group) cohorts. The variables included age, sex, systolic blood pressure (SBP), brachial-ankle pulse wave velocity (ba-PWV), ankle-brachial index, height, weight, maximum leg circumference, lower leg circumference ratio, duration of T2DM, Achilles tendon reflex disorder, and the hemoglobin A1c level. Multiple regression analysis was performed with ba-PWV as the dependent variable and the interaction term between leg circumference ratio and T2DM as the independent variable. The control variables included leg circumference ratio, T2DM, SBP, Achilles tendon reflex disorder, age, and sex. IBM SPSS Statistics for Windows, Version 23.0 (Released 2015; IBM Corp., Armonk, NY, USA) was used for the statistical analysis, with the significance level set to p < 0.05.

Results

The interaction term between the lower leg circumference ratio and T2DM (β = -0.17, 95% CI: -46.11 to -10.92; p < 0.01) was significantly associated with ba-PWV (AdjR² = 0.51, variance inflation factor <4.12). Simple slope analysis indicated that a decreased lower leg circumference ratio was significantly associated with an increased ba-PWV (β = -0.20, p < 0.05) in the T2DM group. No significant relationship was found in the N group (β = -0.03, p = 0.69).

Conclusion

A significant interaction was found between the lower leg circumference ratio and T2DM presence, indicating an association between ba-PWV and the leg circumference ratio specific to patients with T2DM. This result suggests that the leg circumference ratio can be substituted for the ba-PWV to evaluate arterial stiffness in T2DM.

## Introduction

Type 2 diabetes mellitus (T2DM) causes vascular endothelial dysfunction, atherosclerotic diseases, and microvascular disorders [1,2]. Preventing these conditions requires continuous monitoring of arterial stiffness. The most widely used measure to assess arterial stiffness is pulse wave velocity (PWV) [3]. The brachial-ankle PWV (ba-PWV) can predict cardiovascular events [4-6]; greater ba-PWV values predict more severe events. However, measurement requires specialized and expensive equipment; facilities where patients with T2DM live often do not have testing equipment, making evaluation difficult. Facilitating testing for arterial stiffness in patients with T2DM in these settings would improve treatment.

Among the complications of T2DM, diabetic peripheral neuropathy (DPN) appears relatively early in the progression of atherosclerosis [7]. Van et al. reported that DPN in patients with T2DM affects instantaneous muscle force and muscle endurance, with a particularly noticeable effect on the calf muscles of the dorsal lower leg [8].

DPN also affects skeletal muscles. The blood vessels nourishing the muscles on the dorsal surface of the human legs are anatomically distinctive. In the legs’ dorsal sciatic nerve region, muscles and nerves share the same vessels; the vessels penetrate the muscle and nourish the nerve [9,10]. Training the muscles attached to the back of the lower leg increases the conductance [11,12] and size [13] of these blood vessels, suggesting a link between muscle activity and blood vessel dysfunction. When muscles attached to the dorsal surface of the lower leg become dysfunctional, blood flow to the nerves is impaired. T2DM reduces the dynamic response of vascular conductance during exercise [14], suggesting that preserving muscle function is important for maintaining neural function, particularly in patients with T2DM.

Zuo et al. [15] used T2-star (T2*)-weighted MRI of the lower legs of healthy participants with T2DM to investigate the contrast between the soleus and other muscles. The soleus muscle in T2DM showed less T2* luminance and greater deoxyhemoglobin levels than other muscles. This finding suggests that less perfusion occurs in the soleus microvascular bed of patients with T2DM, and it is susceptible to oxidative stress. Because the soleus muscle is rich in mitochondria and capillaries, it readily consumes oxygen for the energy metabolism of slow-twitch muscle fibers [16]. Rats with artificially induced T2DM have reduced capillaries that support red blood cell perfusion [17,18]. In addition, impaired perfusion of the soleus muscle correlates with increased hemoglobin A1c (HbA1c) levels [19].

Because lower leg circumference values are related to the basal metabolic rate [20] and frailty [21], they correspond to part of the general condition. Because T2DM decreases the dynamic response of vascular conductance during exercise [14] and the soleus muscle of patients with T2DM experiences oxidative stress [15], we believe that the lower leg of patients with T2DM will show greater atrophy, even compared to patients in a frail state. Therefore, this study used the circumference of the lower leg as an anthropometric index specific to patients with T2DM, especially skeletal muscle mass.

Atherosclerosis tends to progress peripherally and is more pronounced near the sciatic nerve. The muscles on the back of the lower leg of patients with T2DM are prone to muscle weakness, partly because of DPN. Moreover, the soleus muscle is characterized by abundant muscle fibers, possibly predisposing it to muscle atrophy. Thus, in patients with T2DM, the evaluation of the soleus muscle may be a surrogate indicator of atherosclerosis.

An association has been found between lower leg circumference and ba-PWV [22]. However, whether the association between lower leg circumference and ba-PWV is specific to patients with T2DM is unclear. Therefore, this study aimed to clarify the relationship between arterial stiffness and leg circumference in patients with T2DM compared to participants without T2DM.

## Materials and methods

Participants

This observational study combined data from two studies conducted in the Kanto area to create an archive of patients with type 2 diabetes (T2DM group) and a group without type 2 diabetes (N group). The archives included 227 individuals.

This study was conducted in accordance with the principles of the Declaration of Helsinki. An opt-out option was provided to allow patients and participants to decline participation in the study. This study was approved by the Research Ethics Review Board of the Tokyo Metropolitan Institute of Gerontology (approval no. R24-010, May 31, 2024).

The T2DM group included 132 patients with T2DM from a study of patients attending two internal medicine clinics in Saitama Prefecture. This study was approved by the Ethics Review Committee of the Dokkyo Medical University Nikko Medical Center (Usefulness of screening for pathological progression by measuring lower limb circumference in patients with diabetes; approval no. Nikko30015; July 10, 2020).

Group N enrolled 95 participants with no history of diabetes in a study conducted among older people and others living in the community. This study was approved by the Research Ethics Review Committee of the Tokyo Metropolitan Institute of Gerontology and Geriatric Medicine (Study of short- and long-term effects of a workbook-based picture book reading intervention program; approval no. 1924; June 10, 2019). The study was part of a municipal project conducted in cooperation with a local government in Tokyo, Japan. Participants were recruited through advertisements in community newspapers and letters published by the local government in 2021. Because it was an intervention study, the archive was populated with pre-intervention data.

Variables used

The following variables were included in the T2DM and N groups: age (years), sex (1 = male, 0 = female), systolic blood pressure (SBP, mmHg), ba-PWV (cm/s), ankle-brachial index (ABI), height (cm), weight (kg), BMI (kg/m²), maximum lower leg circumference (cm), lower leg circumference at tibial rough surface height (cm), and medical history.

ABI was not used in the data analysis because it was used as a criterion to exclude samples with ba-PWV errors due to arteriosclerosis obliterans. Because the maximum lower leg circumference is influenced by body size, the lower leg circumference ratio [22] was calculated by dividing the tibial rough surface height by the lower leg circumference. The following variables were included in the T2DM group only: years since the date of diagnosis, presence of DPN, HbA1c level (%), and medication status.

DPN was assessed using two preconditions and three neurological examination items. The preconditions were a diagnosis of diabetes and the ability to rule out neurological diseases other than DPN. DPN was defined as the presence of two or more of the following criteria: symptoms believed to be caused by DPN, reduced vibration thresholds at the bilateral medial malleolus, and decreased or disappearance of the bilateral Achilles tendon reflex [23].

The surveys were conducted between August 2019 and September 2020. The two surveys archived in this study fall under the jurisdiction of different organizations. The authors developed the inspection protocols, and the inspectors were consistently trained by the authors prior to conducting the data collection. This ensured that data collection was conducted with consistent protocols.

Statistical analysis

First, variables for the overall, T2DM, and N groups were described. Second, the correlation coefficients between ba-PWV (cm/s) and each variable in the T2DM and N groups were calculated using Pearson’s product-moment and Spearman’s rank correlation coefficients.

The ba-PWV was used as the dependent variable for all participants. The interaction term between the leg circumference ratio and the presence or absence of T2DM was used as the independent variable. The adjustment variables were the leg circumference ratio, presence or absence of T2DM, SBP (mmHg), disorder of the Achilles tendon reflex, sex (1 = male, 0 = female), and age (years). The independent and adjusted variables were standardized by transforming them into Z-values before being input into the model. If the multiple regression analysis showed that the interaction term between the leg circumference ratio and the presence or absence of T2DM was significantly associated with ba-PWV, the interaction was examined in more detail by simple slope analysis by dividing the leg circumference ratio into two groups with ±1 standard deviation.

Originally, DPN could not be determined by a disorder of the Achilles tendon reflex alone. However, considering that ba-PWV correlates with several variables related to peripheral neuropathy, the disorder of the Achilles tendon reflex was operationally defined as a variable representative of DPN. Statistical analysis was performed using IBM SPSS Statistics for Windows, Version 23.0 (Released 2015; IBM Corp., Armonk, NY, USA) with statistical significance set at p < 0.05.

## Results

Data from two studies were archived for a total of 227 patients. A total of 18 patients were excluded from the study of diabetic patients attending an internal medicine clinic. Five were excluded because of missing ba-PWV data, seven because of missing SBP data, and six because of low ABI (<0.9). Of the 18 subjects excluded from the T2DM study, seven have leg edema. However, these seven overlap with the 18 already excluded, so there are no subjects with leg edema in the analysis population.

A total of five patients were excluded from studies conducted on community-dwelling elderly and other patients. Four were excluded because they had missing ba-PWV data, and one had a low ABI (<0.9).

Prior to enrollment in the study, 12 subjects with a history of T2DM were included in a study conducted on the elderly and other subjects living in the community. However, this study did not enroll subjects with T2DM beforehand. Therefore, the study did not include subjects with T2DM among those who were excluded from the study of community-dwelling elderly and others.

The reason why the data did not include 12 patients with a history of T2DM was that the study from which this data was extracted included elderly people living in the community and did not include information such as HbA1c, years of illness, and medication status.

**Figure 1:**
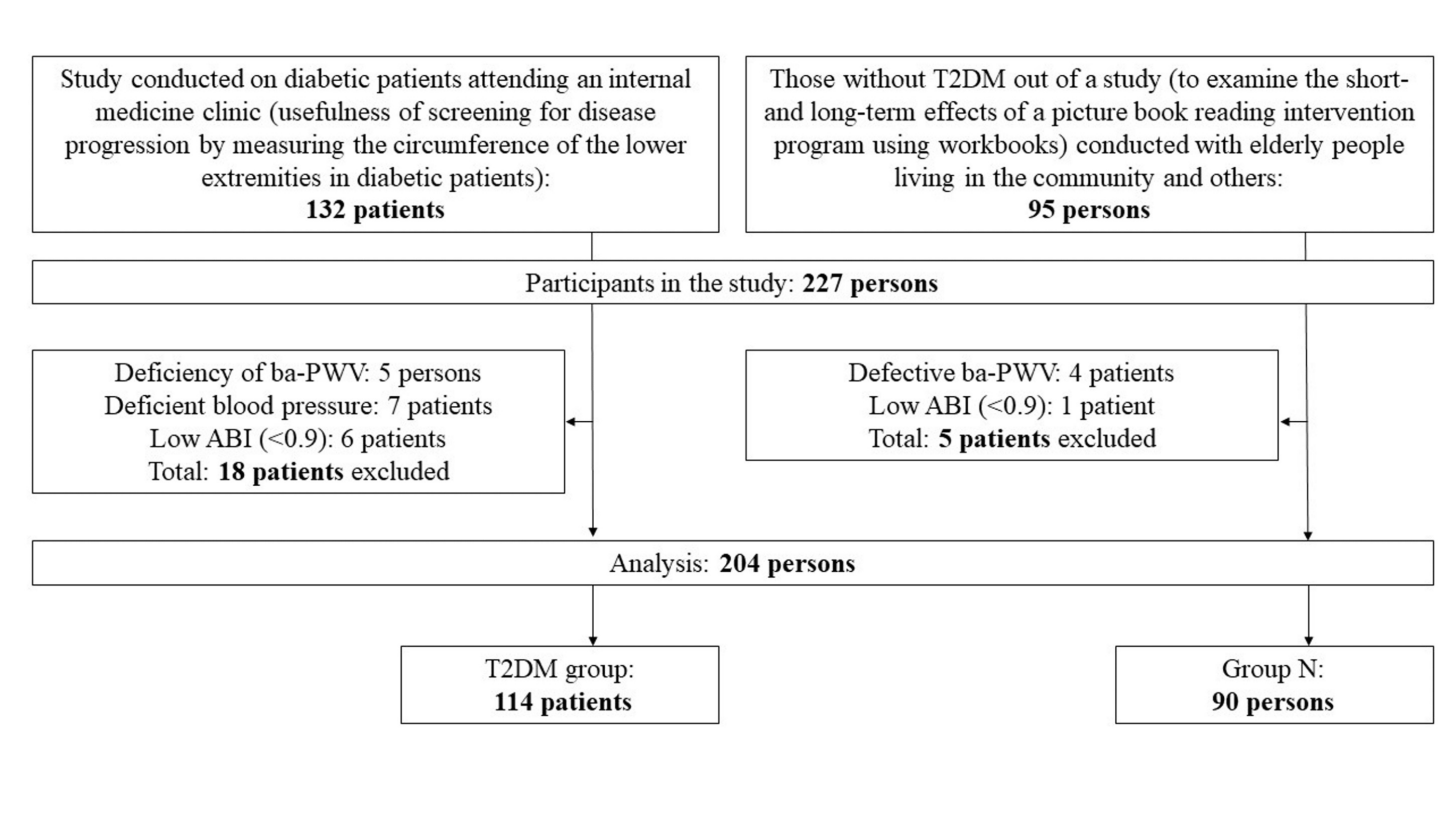
Flow of analysis target extraction ABI, ankle-brachial index; ba-PWV, brachial-ankle pulse wave velocity; T2DM, type 2 diabetes mellitus

A total of 23 subjects were excluded from the two studies, leaving a population of 204 subjects. All subjects were then divided into the DM group (n = 114) and the N group (n = 90) according to the presence or absence of T2DM. The flow of the analysis is shown in Figure 1.

The analysis included 204 subjects, comprising 114 in the DM group and 90 in the N group. The cohort consisted of 73 men and 131 women, with an average age of 66.48 ± 10.53 years. The average SBP was 123.60 ± 23.35 mmHg, while the ba-PWV measured 1,666.90 ± 354.41 cm/s. The ABI averaged 1.39 ± 3.78, and the BMI was 24.06 ± 4.20 kg/m². The lower leg circumference ratio was 1.06 ± 0.05.

Data collected in the T2DM group only showed a duration of T2DM of 11.25 ± 9.45 (years), HbA1c 6.90 ± 0.72 (%), and the T2DM group included 69 DPN patients, of whom 17 had subjective symptoms that could be attributed to DPN, 55 had Achilles tendon reflex disorder, and 74 had inner ankle vibration sensory disorder.

Descriptive results for all subjects and for the DM and N groups are shown in Table 1.

**Table 1:** Descriptive results for all subjects and for the DM and N groups ABI, ankle-brachial index; ba-PWV, brachial-ankle pulse wave velocity; DPN, diabetic neuropathy; HbA1c, hemoglobin A1c; N, not T2DN; SBP, systolic blood pressure; T2DM, type 2 diabetes mellitus

Variable	Unit	Total (n = 204)		T2DM group (n = 114)		N group (n = 90)	
		Total	Mean value ± SD	Total	Mean value ± SD	Total	Mean value ± SD
Age	Years old	‐	66.48 ± 10.53	‐	63.12 ± 11.96	‐	70.73 ± 6.19
Male	People	73	‐	69	‐	4	‐
SBP	mmHg	‐	123.6 ± 23.35	‐	139.36 ± 16.52	‐	103.64 ± 13.27
ba-PWV	cm/s	‐	1,666.9 ± 354.41	‐	1,666.03 ± 368.19	‐	1,668 ± 338.18
ABI	‐	‐	1.13 ± 0.08	‐	1.12 ± 0.08	‐	1.13 ± 0.07
BMI	㎏/m²	‐	24.06 ± 4.2	‐	25.58 ± 4.35	‐	22.13 ± 3.09
Leg circumference ratio	‐	‐	1.06 ± 0.05	‐	1.07 ± 0.05	‐	1.05 ± 0.04
Duration of T2DM	Years	‐	‐	‐	11.25 ± 9.45	‐	‐
HbA1c	%	‐	‐	‐	6.9 ± 0.72	‐	‐
DPN	People	‐	‐	69	‐	0	‐
symptoms possibly due to DPN	People	‐	‐	17	‐	0	‐
Disorder of the Achilles tendon reflex	People	‐	‐	55	‐	0	‐
Disorders of the vibratory sensation of the inner ankles	People	‐	‐	74	‐	0	‐

In addition, the medication status of the T2DM group is shown in Table 2.

**Table 2:** Medications in the T2DM group ACE-I, angiotensin-converting enzyme inhibitor; ARB, angiotensin receptor blocker; CCB, calcium channel blocker; DPP-4, dipeptidyl peptidase-4; GLP1, glucagon-like peptide-1; SGLT-2, sodium glucose cotransporter-2; SU, sulfonylurea; T2DM, type 2 diabetes mellitus

Medication	All (n = 114)
	n (%)
DPP4 inhibitor	57 (50)
Alpha glucosidase inhibitor	51 (44.7)
Biguanide (metformin)	38 (33.3)
SGLT2 inhibitor	25 (21.9)
Insulin	20 (17.5)
GLP1 agonist (injection)	11 (9.6)
Rapid-acting insulin secretagogue (glinide)	8 (7.0)
SU	6 (5.3)
Statins	59 (51.7)
Non-statin hyperlipidemic agents	19 (16.7)
ARB	53 (46.5)
CCB	53 (46.5)
ACE-I	3 (2.6)
Other antihypertensive drugs	14 (12.3)

The correlation coefficients between ba-PWV (cm/s) and each variable were examined using Pearson’s product rate correlation coefficient and Spearman’s rank correlation coefficient. The correlation coefficients between ba-PWV and each variable for the entire subject population, the T2DM group, and the N group are shown in Table 3.

**Table 3:** Correlation coefficients between ba-PWV and each variable ba-PWV, brachial-ankle pulse wave velocity; DPN, diabetic neuropathy; HbA1c, hemoglobin A1c; N, not T2DN; SBP, systolic blood pressure; T2DM: type 2 diabetes mellitus

Variable	Units	Total (n = 204)		T2DM group (n = 114)		N group (n = 90)	
		r/ρ	p-value	r/ρ	p-value	r/ρ	p-value
Age	Years old	0.479	p < 0.001	0.569	p < 0.001	0.423	p < 0.001
SBP	mmHg	0.38	p < 0.001	0.542	p < 0.001	0.672	p < 0.001
BMI	kg/m²	-0.254	p < 0.001	-0.446	p < 0.001	0.044	0.678
Leg circumference ratio	-	-0.314	p < 0.001	-0.522	p < 0.001	0.01	0.922
Duration of T2DM	years	‐	‐	0.213	p < 0.05	‐	‐
HbA1c	％	‐	‐	0.181	0.053	‐	‐
DPN	People	‐	‐	0.034	0.719	‐	‐
symptoms possibly due to DPN	People	‐	‐	-0.062	0.511	‐	‐
Disorder of the Achilles tendon reflex	People	‐	‐	0.206	p < 0.05	‐	‐
Disorders of the vibratory sensation of the inner ankles	People	‐	‐	0.137	0.146	‐	‐

The multiple regression analysis yielded the following results: the interaction term between the lower leg circumference ratio and the presence of T2DM showed a β value of -0.17 (95% CI: -46.11 to -10.92, p < 0.01). The presence of T2DM had a β value of -0.60 (95% CI: -563.25 to -284.35, p < 0.001), while the lower leg circumference ratio had a β value of -0.07 (95% CI: -65.43 to 12.78, p = 0.186). SBP exhibited a β value of 0.79 (95% CI: 223.96 to 335.48, p < 0.001). Disorder of the Achilles tendon reflex had a β value of 0.14 (95% CI: 14.81 to 201.02, p < 0.05). Sex had a β value of 0.09 (95% CI: -26.77 to 156.79, p = 0.164), and age showed a β value of 0.30 (95% CI: 62.38 to 150.64, p < 0.001). The detailed results are presented in Table 4.

**Table 4:** Results of multiple regression analysis AdjR² = 0.51, p < 0.05, variance inflation factor <4.12 Leg circumference ratio × presence of T2DM: interaction term between leg circumference ratio and the presence of T2DM SBP, systolic blood pressure; T2DM, type 2 diabetes mellitus

	β	95％ CI		p-value
		Lower limit	Upper limit	
Leg circumference ratio x presence of T2DM	-0.17	-46.11	-10.92	p < 0.01
Presence of T2DM	-0.6	-563.25	-284.35	p < 0.001
Leg circumference ratio	-0.07	-65.44	12.78	p = 0.19
SBP	0.79	223.96	335.48	p < 0.001
Disorder of the Achilles tendon reflex	0.14	14.81	201.02	p < 0.05
Sex	0.09	-26.77	156.79	p = 0.16
Age	0.3	62.38	150.65	p < 0.001

**Figure 2:**
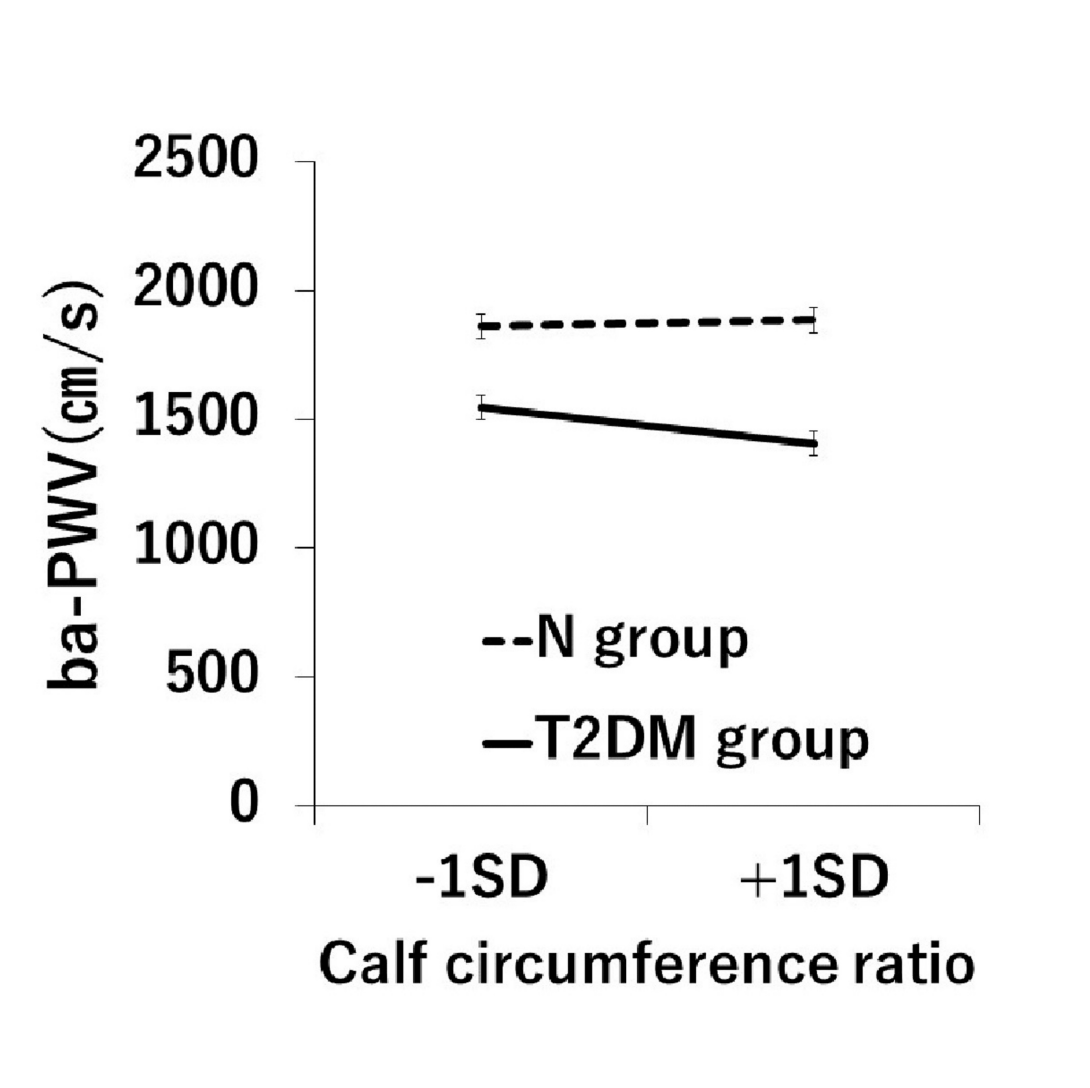
Results of simple slope analysis ba-PWV, brachial-ankle pulse wave velocity

The results of simple slope analysis showed that in the T2DM group, a decrease in the lower leg circumference ratio was statistically significantly associated with an increase in ba-PWV (β = -0.20, p < 0.05). On the other hand, no statistically significant relationship was found in the N group (β = 0.03, p = 0.69). A graphical representation of the results is shown in Figure 2.

## Discussion

This study aimed to clarify the relationship between arterial stiffness and the leg circumference ratio in patients with T2DM. We found that ba-PWV, an arterial stiffness index, correlated negatively with the lower leg circumference ratio only in patients with T2DM. The interaction between ba-PWV and lower leg circumference ratio in the presence of T2DM showed a significant association after adjusting for SBP, Achilles tendon reflex disorder, sex, and age.

DPN was not a statistically significant variable in the correlation between ba-PWV and DPN (as defined by the criteria proposed by Yasuda et al. [23]). Of the three items comprising DPN, only the disorder of the Achilles tendon reflex showed a significant association. The disorder of the Achilles tendon reflex is the only one of the three DPN-defining items that includes motor nerve damage, a symptom related to muscle atrophy. We suggest that DPN was insignificant because, according to the criteria proposed by Yasuda et al. [23], a participant was judged to have DPN even if only items indicating sensory nerve impairment were applicable; thus, participants without motor nerve impairment were considered to have DPN. This rationale is why this study used only the disorder of the Achilles tendon reflex as a DPN-indicating variable to investigate the association between atherosclerosis and muscle atrophy.

In multiple regression analysis, the ba-PWV and lower leg circumference ratio were significantly correlated only in patients with T2DM. The soleus muscle consumes oxygen to support the energy metabolism of slow-twitch muscle fibers; impaired perfusion increases HbA1c levels, suggesting the soleus muscle is more susceptible to oxidative stress in patients with T2DM. From our analyses, we conclude that characteristic muscle atrophy occurs in patients with T2DM. Essentially, ba-PWV values correspond to the arterial stiffening in large vessels. We hypothesize that the impaired capillary perfusion in the lower leg manifests as an increase in the ba-PWV, mediated by the impaired function of the soleus muscle, inducing vascular dysfunction. Thus, the lower leg circumference ratio correlation with ba-PWV corresponds to arterial stiffness.

Limitations

This study had several limitations. First, patients with T2DM were from two internal medicine clinics in Saitama Prefecture; therefore, its generalizability among patients with diabetes is limited. Second, the numbers of men and women differed in the N group. Sex is associated with ba-PWV, with or without T2DM [24]. This imbalance may have affected our results. Third, T2DM is a lifestyle-related disease associated with multiple factors. The multivariate analysis in this study was adjusted for only physical parameters; it did not consider psychosocial factors. This study should be reexamined in the future by expanding the study data to include multiple facilities, increasing the sample size, and collecting psychosocial variables. Fourth, the analysis is not based on the impact of smokers, alcohol, hypertension, and other comorbidities. This was due to the inability to collect a detailed history of medical history in the N group.

## Conclusions

This study aimed to clarify the relationship between arterial stiffness and muscle status in patients with T2DM. It found that the lower leg circumference ratio is associated with arterial stiffness. Multivariate analysis revealed that this association between ba-PWV and the leg circumference ratio is specific to patients with T2DM. These findings suggest that the lower leg circumference ratio could potentially serve as a predictor of arterial stiffness when ba-PWV measurements are unavailable.
